# Necrological

**Published:** 1888-07

**Authors:** 


### NECROLOGICAL

DR. Y. D. HARRINGTON died at his residence in this city June 9, 1888, of follicular enteritis; aged 70 years. He was a native of south Carolina; was born March 18, 1818; moved to Texas at quite an early date, and lived in Rusk county till 1875, where he practiced successfully his profession; was a graduate of Galveston Medical College, and rendered valuable services in the late war. Being a member of the Terrell Medical Society, in good and regular standing, a called meeting was held, and a committee appointed who drafted the following resolutions:

WHEREAS, It has pleased Almighty God, in the exercise of His beneficent providence, to remove from his respective position of usefulness and from his numerous associates in medicine the late Dr. Y. D. Harrington;

WHEREAS, We realize, with a feeling sense, the loss thus sustained, we deem it our sad duty to give expression to so great a loss, and our own appreciation of his worth as a physician and associate; therefore be it

*Resolved*, That the Terrell Medical Society has been deprived of one of its most active supporters, whose thoughtful counsel placed him in the conspicuous position of its members.

*Resolved*, That as individual members we are painfully sensible of this, our loss. As a man he was consistent, thoughtful, honest, just, and an exemplary Christian gentleman, whose character was endowed with tenderness and affection.

*Resolved*, That we hereby tender his bereaved family our sincere sympathy as professional brethren, and invoke that consolation for them which can only be conferred by Hin who doeth all things well.

*Resolved*, That these resolutions be placed upon the minutes of the Terrell Medical Society, and that DANIEL'S TEXAS MEDICAL JOURNAL, *The Times* and *The Star* be requested to publish the same, and that a copy be forwarded the family of the deceased.

DR. JOHN INABNIT,

DR. W. P. DUMAS,

DR. E. E. SMILEY,

Committee.

Terrell, Texas, June 11, 1888.

